# *Toxocara canis* Infection Alters mRNA Expression Profiles of Peripheral Blood Mononuclear Cells in Beagle Dogs at the Lung Infection Period

**DOI:** 10.3390/ani12121517

**Published:** 2022-06-10

**Authors:** Lang Cai, Yang Zou, Yue Xu, Hao-Yu Li, Shi-Chen Xie, Xing-Quan Zhu, Wen-Bin Zheng

**Affiliations:** 1 Laboratory of Parasitic Diseases, College of Veterinary Medicine, Shanxi Agricultural University, Jinzhong 030801, China; cai458334979@163.com (L.C.); xuyue315217@163.com (Y.X.); haoyuli1@126.com (H.-Y.L.); xieshichen221@163.com (S.-C.X.); 2 State Key Laboratory of Veterinary Etiological Biology, Key Laboratory of Veterinary Parasitology of Gansu Province, Lanzhou Veterinary Research Institute, Chinese Academy of Agricultural Sciences, Lanzhou 730046, China; zouyangdr@163.com; 3 Key Laboratory of Veterinary Public Health of Higher Education of Yunnan Province, College of Veterinary Medicine, Yunnan Agricultural University, Kunming 650201, China

**Keywords:** *Toxocara canis*, toxocariasis, Beagle dog, PBMCs, RNA-seq

## Abstract

**Simple Summary:**

Toxocariasis is one of the most neglected zoonoses in the world. *Toxocara canis* is the main pathogen causing toxocariasis in humans and animals, threatening public health. To date, the mechanism by which the larvae of *T*. *canis* escape the attack of immune cells in the blood is still poorly understood. Using RNA-seq technology, the transcriptional alterations of Beagle dog peripheral blood mononuclear cells (PBMCs) between the presence and absence of *T. canis* infection were analyzed during the lung infection period, and 1066 upregulated genes and 1076 downregulated genes were identified (padj < 0.05 and |log_2_ (FoldChange)| > 1). In addition, many immune- or inflammation-related GO terms and KEGG signaling pathways were significantly altered during *T. canis* infection by GO annotation and KEGG enrichment analysis. The present study revealed that *T. canis* infection can alter the mRNA profiles of PBMCs in Beagle dogs during the lung infection period, which has important implications for a better understanding of the interaction mechanism between *T. canis* and host immune cells.

**Abstract:**

*Toxocara canis* is a neglected zoonotic roundworm distributed all over the world, causing toxocariasis in humans and animals. However, so far, the immune mechanism of *T. canis* infection in definitive hosts remains to be clarified. In this study, the transcriptional alterations of Beagle dogs’ peripheral blood mononuclear cells (PBMCs) induced by *T. canis* infection during the lung infection period were analyzed using RNA-seq technology. A total of 2142 differentially expressed genes were identified, with 1066 upregulated genes and 1076 downregulated genes. Many differentially expressed genes participated in the biological process of intracellular signal transduction, as well as the immune- or inflammation-related KEGG signaling pathway, such as the Notch signaling pathway, Toll-like receptor signaling pathway, and NF-kappa B signaling pathway, through KEGG enrichment analysis. This study indicated that *T. canis* infection could suppress the biological function of Beagle dogs’ PMBCs and provided basic data to further clarify the interaction mechanism between *T. canis* and host immune cells.

## 1. Introduction

Toxocariasis, mainly caused by *Toxocara canis*, is a severely neglected zoonotic parasitic disease, and is widespread all over the world [1]. Dogs and other canines are the definitive hosts for *T. canis*, while humans and many animals are paratenic hosts for *T. canis* [2]. Although paratenic hosts are not suitable for *T. canis* to complete the life cycle, the *T. canis* larvae can migrate through multiple tissues, causing significant damage, such as visceral larva migrans (VLM), ocular larva migrans (OLM), and neurotoxocariasis (NT) [2]. Fertilized eggs can be discharged continuously from dogs and other canines infected with *T. canis*, and develop into infective eggs (embryonated eggs) under suitable conditions [3]. Humans and animals can become infected by *T. canis* by consuming food/water contaminated with infective eggs, and *Toxocara* eggs are very common in public places, with a prevalence of 21% [4,5]. The prevalence of *T. canis* was 19% and 12.14% to 44.83% in humans in the world and mainland China, respectively [6,7], and for dog toxocariasis, it was 11.1% and 17.34%, respectively [8,9]. Therefore, controlling *T. canis* infection in dogs is extremely important for the prevention and control of humans’ or animals’ toxocariasis.

After embryonated eggs are hatched in the host small intestine, the L3 larvae penetrate the intestinal wall, enter the bloodstream, and reach the liver; subsequently, they are transported to the lung at 96 h post-infection (hpi), which is the lung infection period of *T. canis* in hosts [10]. In the lung, the L3 larvae begin to undergo two completely different migratory pathways in definitive hosts and paratenic host [10]. In the definitive hosts, the L3 larvae can penetrate the pulmonary alveoli and reach the trachea, which is the prerequisite route for L3 larvae to reentry into the gastrointestinal tract, while in the paratenic hosts, through the passive transport of the pulmonary artery, they migrate to various tissues, where they are arrested at the L3 stage for extensive periods [10]. This phenomenon raised a question as to what role immune cells of the host’s blood play during the stage of the pulmonary phase of *T. canis* infection. Infection of *T. canis* can significantly alter the immune system, and it has been shown that eosinophils, macrophages, and lymphocytes can be clustered during lung and liver infection [11]. Under the influence of the immune system, the pathogenesis caused by *T. canis* infection mainly depends on the dynamic balance of pro- and anti-inflammatory responses, and the pathology of toxocariasis mainly results from the imbalance of pro- and anti-inflammatory cytokines in patients [12]. Peripheral blood mononuclear cells (PBMCs), including lymphocytes, monocytes, and macrophages, are important immune cells that can play critical biological functions, such as maintaining host homeostasis and defending against parasite infection [11,13]. Thus, understanding the transcriptional response of PBMCs during *T. canis* infection can help to reveal the regulation mechanism of the host immune system against *T. canis* and to formulate effective prevention and control strategies for toxocariasis.

The continuous development of next-generation sequencing technologies, such as genomics, transcriptomics, and proteomics, has contributed to the study of the molecular biology of *T. canis* [14,15]. Analysis of mRNA expression profiles may reveal novel molecular mechanisms based on the pathophysiology of *T. canis* and search for novel gene targets for drug development. In this study, RNA-seq was conducted to reveal transcriptional alterations in the PBMCs of Beagle dogs during the lung infection period of *T. canis*. Our study provides basic data for identifying the role of host PBMCs and highlights the role of potential signaling pathways in the pulmonary infection phase of *T. canis* infection, particularly the immune and inflammation pathways.

## 2. Materials and Methods

### 2.1. Ethics Approval

This study was approved by the Animal Administration and Ethics Committee of Lanzhou Veterinary Research Institute, Chinese Academy of Agricultural Sciences (Permit No: 2018-015). The Beagle dogs used in this study were handled in accordance with good animal practices required by the Animal Ethics Procedures and Guidelines of the People’s Republic of China.

### 2.2. Animal and Sample Collection

Six experimental SPF Beagle dogs from one litter, 6–7 weeks old, were purchased from and housed at the National Canine Laboratory Animal Resource Center (Guangzhou, China). All puppies were fed three times daily with free access to water at 24 °C. They were randomly divided into an infection group (n = 3) and a control group (n = 3). After one week of breeding to adapt to the laboratory environment, the puppies in the infection group were infected orally with 300 infective eggs in 1 mL of normal saline, and the puppies in the control group were mock-infected orally with equal amounts of normal saline [16]. The infective eggs of *T. canis* were collected as in our previously described method [17]. Briefly, fertilized eggs were obtained from the uteri of adult female *T. canis*, and then incubated on filter papers with 0.5% formaldehyde solution at 28 °C with 85–95% relative humidity for 28 days. The infective eggs were obtained and stored in 1% formaldehyde solution at 4 °C. At 96 hpi, blood was collected from the jugular vein of six puppies and placed into tubes with EDTA-K_2_ to conduct the complete blood count by an automated blood analyzer (XT2000 iv; Sysmex, Kobe, Japan) and to separate PBMCs by Dogs PBMCs Isolate kit (TBDscience, Tianjin, China). PBMCs were stored at −80 °C for RNA extraction. The puppies were then humanely euthanized with 0.75 mg/kg 10% KCl under a general anesthetic treatment with 10 mg/kg Zoletil 50 (Virbac, Nice, France) to collect the liver and lung tissues to recover *T. canis* [18].

### 2.3. RNA Extraction and RNA Sequencing Analysis

The total RNA of PBMC was extracted using TRIZOL (Life Technologies, Carlsbad, CA, USA), and genomic DNA was removed from total RNA using DNase I (NEB, Ipswich, MA, USA). RNA integrity was assessed using a Bioanalyzer 2100 system (Agilent Technologies, Santa Clara, CA, USA). Each mRNA was purified from 1 μg total RNA using poly-T oligo-attached magnetic beads to synthesize cDNA. After adenylating the 3′ ends of cDNA fragments into blunt ends by exonuclease/polymerase activities, the adaptor with a hairpin loop structure was ligated. The library fragments were purified on an AMPure XP system (Beckman Coulter, Beverly, CA, USA) and amplified using universal PCR primers and index (X) primer. Then PCR products were purified on the AMPure XP system, and library quality was assessed on the Agilent Bioanalyzer 2100 system. Subsequently, the libraries were sequenced utilizing the Illumina HiSeq 2500 Platform, and 150 bp paired-end reads were generated.

The original raw reads with adaptor and reads containing uncertain base information, and low quality reads with a base number of Qphred ≤ 20 exceeding 50% of the entire read length were removed to obtain high-quality clean reads for downstream analyses. In addition, the Q20, Q30, and GC content of the clean data were calculated to estimate the data quality [19]. The RNA-seq data were mapped to the reference genome of *Canis lupus familiaris,* downloaded from the Ensembl database (release-99) by Hisat2 v2.0.5 [20]. The mapped reads were assembled using StringTie (v1.3.3) [21]. FeatureCounts v1.5.0-p3 was used to count the read numbers mapped to each gene. The FPKM of each gene was calculated based on the length of the gene and the read count mapped to this gene. Differential expression analysis between the infection group and the control group was conducted using the DESeq2 R package (1.20.0). Genes with an adjusted *p*-value (padj) < 0.05 and |log_2_ (FoldChange)| > 1 were assigned as differentially expressed (DE).

### 2.4. GO Annotation and KEGG Pathway Enrichment Analysis

Gene Ontology (GO) annotation and Kyoto Encyclopedia of Genes and Genomes (KEGG) pathway enrichment analysis of differentially expressed mRNAs (DEmRNAs) were conducted using the clusterProfiler R package (v 3.4.4) [22]. GO and KEGG terms with *p*-value < 0.05 were considered significantly enriched.

### 2.5. Quantitative Real Time RT-PCR (qRT-PCR)

The expression level of the 10 selected DEmRNAs was analyzed by qRT-PCR to confirm the results of RNA-seq. The qRT-PCR experiment was performed on LightCycler480 (Roche, Basel, Switzerland), and commercial kits, amplification conditions, and melting curve analysis for DEmRNA amplification were used, as described previously [16]. Briefly, a PrimeScript™ RT reagent kit with gDNA (genomic DNA) Eraser (Takara, Tokyo, Japan) was used to synthesize the first-strand cDNA of mRNA. An EvaGreen qPCR MasterMix-no dye qRT-PCR Kit (abm, Zhenjiang, China) was used for the mRNA amplification. The conditions of mRNA amplification were 95 °C for 10 min, 40 cycles of 94 °C for 15 s, and 60 °C for 1 min. Melting curve analysis was performed to ensure specific amplification in each reaction using the following conditions: 95 °C for 10 s, 65 °C for 1 min, and a progressive increase from 65 °C to 95 °C. The expression level of the selected DEmRNAs was normalized through the housekeeping gene. The primers are listed in Table S1. 

## 3. Results

### 3.1. Confirmation of T. canis Infection and Overview of RNA Sequencing

**Figure 1:**
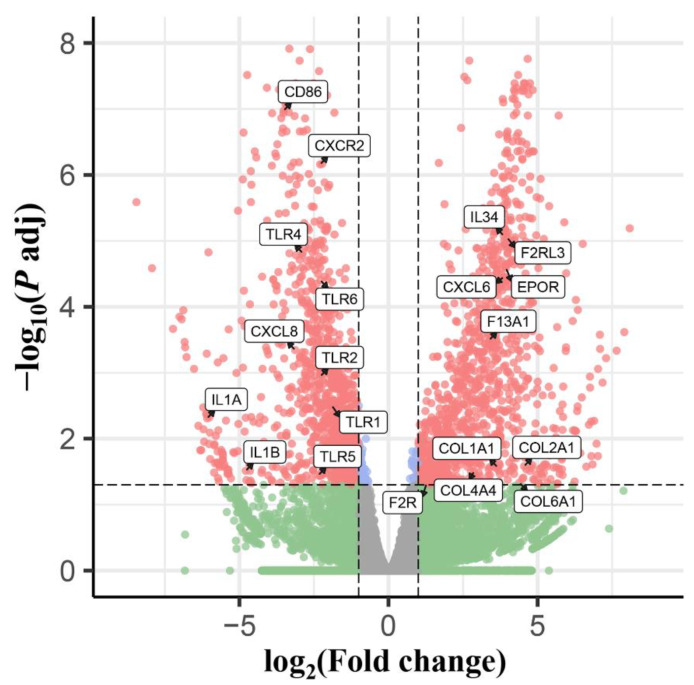
The volcano plots showing the differentially expressed transcripts identified from the PBMCs of puppies at 96 h after infection with 300 *Toxocara canis* eggs (padj < 0.05 and |log_2_ (FoldChange)| > 1).

**Figure 2:**
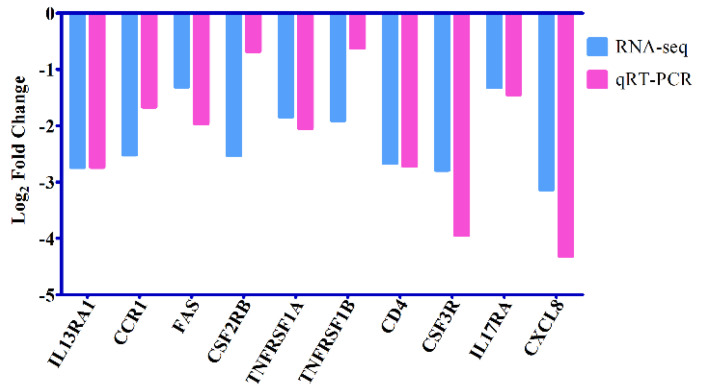
Verification of the expression of 10 selected differentially expressed mRNAs (DEmRNAs). The Y-axis denotes the log_2_ fold change, and the X-axis shows the analyzed DEmRNAs.

The phenomenon of eosinophilia was observed at 96 hpi, and the larvae of *T. canis* were found in the livers and lungs of all infected puppies, with an average of 21 and 27.3 larvae, respectively, which was reported in detail in our previous study [23]. For the RNA-seq data, 272,757,414 raw reads and 265,042,710 high-quality clean reads were generated from six RNA libraries, and the error rate of the reads was 0.03. The average Q20, Q30, and GC content was 97.5%, 93.4%, and 50.3%, respectively (Table 1). A total of 21,169 transcripts were identified in this experiment, and 2142 differentially expressed transcripts were identified between dogs infected by *T. canis* and uninfected dogs, among which 1076 transcripts were upregulated and 1066 transcripts were downregulated, such as TLR1/2/4/5/6, CXCL6/8 and IL1A/1B (Figure 1 and Table S2). The qRT-PCR results of the expression level of 10 selected DEmRNAs were consistent with the RNA-seq results in the trend and magnitude (Figure 2), confirming the validity of the RNA-seq data in the present study. The raw data for RNA-seq analysis has been submitted to the NCBI Sequence Read Archive (SRA) under accession number PRJNA806788.

### 3.2. GO Annotation and KEGG Pathway Enrichment Analysis

**Figure 3:**
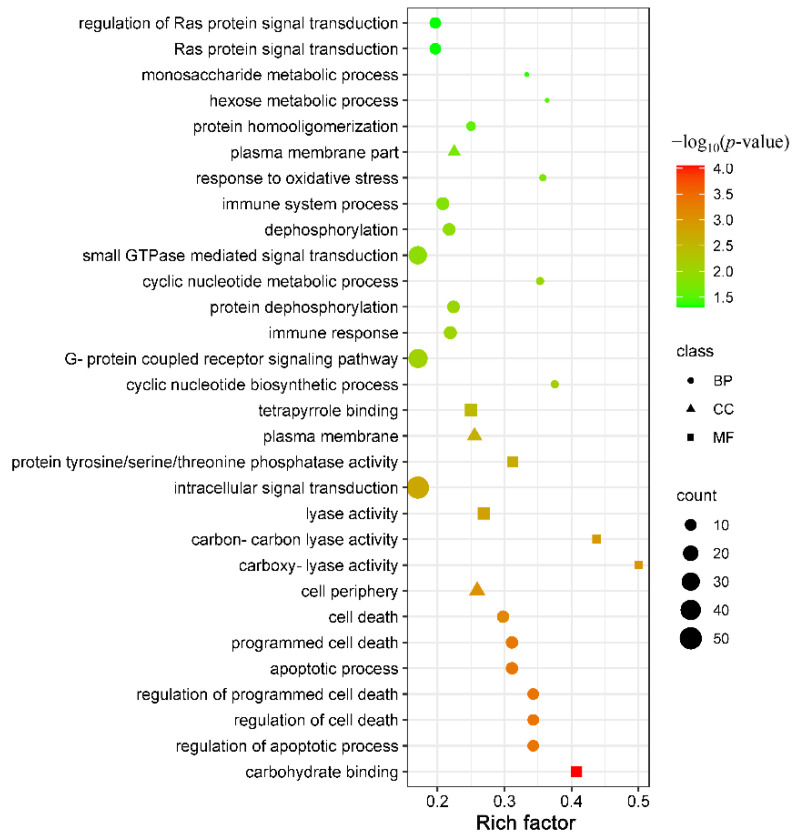
The enrichment score, *p*-value, count, and class of the top 30 differentially expressed GO terms. The X-axis label represents the rich factor, and the Y-axis label shows the GO terms. The rich factor reflects the proportion of differentially expressed mRNAs (DEmRNAs) in a given GO term. The greater the rich factor, the greater the degree of term enrichment. The color of the dots represents the enrichment score [−log_10_(*p*-value)], where red indicates high enrichment and green indicates low enrichment. Dot size represents the number of DEmRNAs in the corresponding term (bigger dots indicate larger DEmRNA numbers).

**Figure 4:**
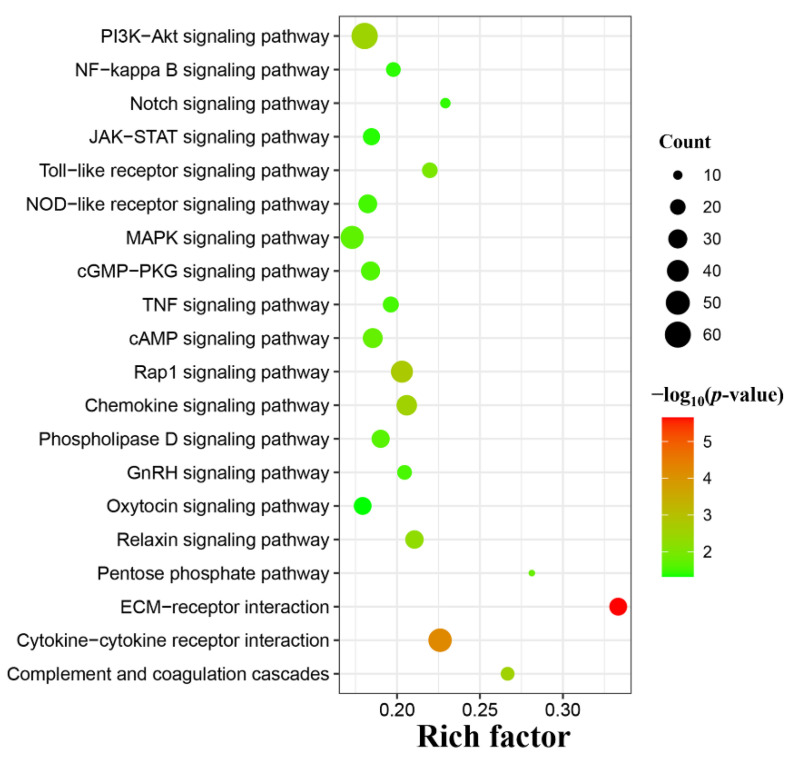
Classical signaling pathways were identified in this study. The X-axis label represents the rich factor, and the Y-axis label shows the KEGG pathways. The rich factor reflects the proportion of differentially expressed mRNAs (DEmRNAs) in a given pathway. The greater the rich factor, the greater the degree of pathway enrichment. The color of the dots represents the enrichment score [−log_10_(*p*-value)], where red indicates high enrichment and green indicates low enrichment. Dot size represents the number of DEmRNAs in the corresponding pathway (bigger dots indicate larger DEmRNA numbers).

In the present study, a total of 310 DEmRNAs with 118 upregulated mRNAs and 192 downregulated mRNAs were significantly enriched in 54 GO terms at 96 hpi, including 21 terms of biological process, 3 terms of cellular component, and 30 terms of molecular function. The number of downregulated mRNAs was greater than that of the upregulated mRNAs in the majority of differentially enriched GO terms (64.81%, 35/54). In addition, many differentially enriched GO terms were involved in the immune response, such as chemokine receptor binding, immune response, and cytokine activity (Table S3). The top 30 significantly enriched GO terms are shown in Figure 3. A total of 507 DEmRNAs with 195 upregulated mRNAs and 312 downregulated mRNAs were significantly enriched in 69 KEGG signaling pathways at 96 hpi, and the number of downregulated mRNAs was greater than that of the upregulated mRNAs in the majority of differentially enriched KEGG pathways (75.36%, 52/69) (Table S4). In addition, many differentially enriched classical signaling pathways associated with immunity and inflammation were identified in this study, such as the PI3K-Akt signaling pathway (61/388), Toll-like receptor signaling pathway (20/91), MAPK signaling pathway (46/266), Notch signaling pathway (11/48), NF-kappa B signaling pathway (18/91) and JAK-STAT signaling pathway (24/130) (Figure 4). Intriguingly, many ECM constituents were significantly altered in PBMCs after *T. canis* infection, such as the upregulation of collagens (including COL1A1, COL2A1, COL4A3, COL4A4, and COL6A1), laminin (LAMC1), and thrombospondin (THBS3), as well as the downregulation of fibronectin (FN1) and osteopontin isoform X2 (SPP1). Moreover, in this study, many integrin genes and proteoglycan were upregulated, such as IGTA2, IGTB1, IGTB3, and CD44 (Figure S1).

## 4. Discussion

The mRNA expression profiles of Beagle dogs’ PBMCs were investigated between dogs infected by *T. canis* and uninfected dogs at 96 hpi based on the RNA-seq technique to improve the understanding of toxocariasis pathogenesis. The present study showed that the number of upregulated mRNAs (1076) was similar to that of downregulated mRNAs (1066). However, the bioinformatics analysis of GO annotation and KEGG pathway enrichment showed that the biological function of the puppies PBMCs may be comprehensively suppressed after *T. canis* infection with the number of downregulated mRNAs was observably greater than that of the upregulated mRNAs in the majority of differentially enriched GO terms (64.81%, 35/54) and KEGG pathways (75.36%, 52/69), especially in some immune- or inflammation-related GO terms or KEGG signaling pathways. This study revealed that *T. canis* could regulate the host immune response by affecting the biological function of host PBMCs through the Notch signaling pathway, Toll-like receptor signaling pathway, and ECM-receptor interaction pathway, by which *T. canis* evades host immune monitoring or attack [24]. We have previously reported a detailed global profiling of lncRNAs-miRNAs-mRNAs of Beagle dogs’ lungs following *T. canis* infection at 24 hpi, 96 hpi, and 36 days post-infection (dpi), which indicated that the pathogenesis of toxocariasis in the lung is mediated through contributions from pro- and anti-inflammatory mechanisms [16]. In addition, 264 DEmRNAs were identified in the Beagle dogs lung between dogs infected by *T. canis* and uninfected dogs at 96 hpi in previous lung transcriptome analysis [16], which was far lower than the number of DEmRNAs identified in PBMCs in this study, indicating that infection had a greater effect on PBMCs than lung at the lung infection period of *T. canis*.

Of the 54 significantly enriched GO terms, many were related to signal transduction, such as intracellular signal transduction (57/334) with 17 upregulated mRNAs and 40 downregulated mRNAs, small GTPase-mediated signal transduction (36/211) with 7 upregulated mRNAs and 29 downregulated mRNAs, and Ras protein signal transduction (12/61) with 1 upregulated mRNAs and 11 downregulated mRNAs. Through intracellular signal transduction, cells can convert information acquired from the extracellular milieu into chemical stimuli to react. A previous study showed that phagocytosis of African trypanosomes by macrophages initiates intracellular signal transduction cascades that lead to the release of pro-inflammatory cytokines and alteration in cell function, which contributes to the immunopathogenesis of African trypanosomiasis [25]. The magnitude and quality of intracellular signal transduction significantly affect the outcome of parasite infection, and unlike protozoa, such as trypanosomes, the excretory-secretory products of helminths can disrupt the activation of lymphocytes, macrophages, and dendritic cells by intracellular signal transduction, such as filarial nematodes [26]. Furthermore, some classical pathways associated with intracellular signal transduction were significantly enriched at 96 hpi, such as the cAMP signaling pathway (33/178) with 11 upregulated mRNAs and 22 downregulated mRNAs, the MAPK signaling pathway with 9 upregulated mRNAs and 37 downregulated mRNAs (46/266), cGMP-PKG signaling pathway with 15 upregulated mRNAs and 15 downregulated mRNAs (30/13) and JAK-STAT signaling pathway (24/130) with 8 upregulated mRNAs and 16 downregulated mRNAs. These results suggest that *T. canis* infection could inhibit intracellular signal transduction in puppies’ PBMCs, affecting the host response to *T. canis* infection.

Moreover, two significantly enriched GO terms were associated with the immune process, including immune response and immune system process, with 7 upregulated mRNAs and 9 downregulated mRNAs, such as IL1A, CXCL6/8 and CCL5/14. Moreover, various classical signaling pathways associated with immunity and inflammation were significantly enriched, such as the Notch signaling pathway, Toll-like receptor signaling pathway, and NF-kappa B signaling pathway (Figure 4). The Notch signaling pathway is associated with inflammation and immunity and can contribute to the occurrence and development of inflammation [27,28]. The change in the Notch signaling pathway can cause macrophages to be polarized to the M2 subtype, leading to an anti-inflammatory response [29]. Despite the overall manifestation of the Notch signaling pathway being downregulated (11/48) with 2 upregulated mRNAs and 9 downregulated mRNAs in this study, notch4 and presenilin-2 were found to be upregulated. The expression level of notch4 in dogs infected with *T. canis* was upregulated 11.76 times, which is very notable. Notch4 is an anti-inflammatory gene that plays a negative regulator of macrophage activation by diminishing the expression of pro-inflammatory cytokines, such as IL-6 and IL-12, as well as costimulatory markers, such as CD80 and CD86 [30]. In addition, notch4 seems to inhibit the Toll-like receptor signaling pathway by affecting TAK1 activation [31]. In our study, IL-6, IL-10, and CD86 were evidently downregulated, showing that the up-regulation of notch4 could resist the invasion of *T. canis* by inhibiting the activation of macrophages. So far, little is known about the functions of notch4 in parasite infection. Toll-like receptors are a family of pathogen-associated pattern recognition receptors, which are usually identified in sentinel cells and contribute to the innate immune system. As part of the innate immune response, TLR-induced signaling pathways can play essential roles during parasitic infections, such as eradicating the invading of parasites [32]. The abnormal activation of TLR can cause tissue damage, while the negative regulation of TLRs can attenuate the production of pro-inflammatory cytokines and limit excessive pathology. Previous studies showed that the expression of TLR1/3/7/8 were repressed strongly at 8 weeks post-infection during the course of *Schistosoma* infection, especially TLR3 [33], and the expression of TLR1/2/4/9 were decreased on B cells and monocytes after filarial infection [34,35]. In this study, the interesting phenomena were the significant downregulation of the Toll-like receptor signaling pathway (20/91) with 1 upregulated mRNA and 19 downregulated mRNAs, including the downregulation of the transcription level of TLR1/2/4/5/6, MYD88, IRAK4 and IL-1β (IL-1B). This was also similar to a previous report that *Fasciola hepatica* infection can comprehensively suppress Toll-like receptor expression in ovine PBMCs at the acute infection stage, leading to downregulation of the transcription level of TLR1/5/67/10 in ovine PBMC [36]. IL-1 is closely linked to immune and inflammation regulation, such as inducing prostaglandin production through cyclooxidase-2, producing nitric oxide by increasing levels of inducible nitric oxide synthase, and inducing the expression of many cytokines, which play a critical role in resisting and killing parasites in host bodies [37]. IL-1β is one of the main cytokines in IL-1 molecules, and is an important pro-inflammatory cytokine involved in the innate immunity response [38]. The excretory-secretory products of *T. canis* can induce the upregulation of IL-1β in THP-1 macrophages in vitro [12], while, the expression level of IL-1β was evidently downregulated 27.62 times between dogs infected by *T. canis* and uninfected dogs in this study, suggesting that *T. canis* could escape the attack of the host immune system by inhibiting IL-1β production *in vivo*. The role of notch4 and IL-1β in PBMCs still merits further exploration during *T. canis* infection.

Another interesting phenomenon was the significantly altered ECM-receptor interaction pathway (27/78) that is a micro-environmental pathway that maintains cell and tissue structure and function [39]. The ECM consists of a complex mixture of structural and functional macromolecules, including collagen, fibronectin, and laminin, which form specific interactions with cells through transmembrane receptors, such as integrins and proteoglycans [40]. In this study, 21 upregulated mRNAs and 5 downregulated mRNAs in the ECM-receptor interaction pathway were identified between dogs infected by *T. canis* and uninfected dogs, including many upregulated ECM constituents, integrin genes, and proteoglycan, suggesting that *T. canis* infection can activate the ECM-receptor interaction pathway, which could provide valuable resources for further exploring the interaction mechanism of *T. canis* hosts. Integrin genes and proteoglycan can promote cell migration by participating in cell adhesion and cell-surface-mediated signaling between the ECM and cells [41], such as ITGA6 and CD44, which were upregulated 14.12 times and 3.68 times in this study. Another significant change was the upregulation of collagen in this study. Collagen, a key component of the ECM, contributes to the mechanical strength and elasticity of tissues and acts as a natural substrate for cellular attachment, proliferation, and differentiation, playing critical roles in the regulation of the phases of wound healing, either in its native, fibrillar conformation or as soluble components in the wound milieu [42]. The invasion of *T. canis* has both physical and chemical effects on the hosts, and wound healing is crucial to repairing the damage induced by the invasion of *T. canis* in hosts. Therefore, the upregulation of collagen expression may be attributed to wound healing after *T. canis* migration. Overall, the present study revealed that *T. canis* infection could suppress intracellular signal transduction, as well as the immune and inflammatory responses of puppies’ PBMCs through multiple signaling pathways, affecting the host response to *T. canis* infection. Meanwhile, the migration and cell adhesion of PBMCs could be enhanced by the activation of the ECM–receptor interaction pathway.

## 5. Conclusions

In this study, we investigated the mRNA expression profiles of PBMCs in Beagle dogs infected with *T. canis* at 96 hpi. The results showed that *T. canis* infection can alter many genes related to immunity and inflammation in PBMCs, and that these altered genes may suppress the biological function of puppies’ PBMCs. This result provided a valuable resource for understanding the interaction mechanism between *T. canis* and its host immune cells.

## Figures and Tables

**Table 1:** Overview of RNA sequencing data in this study.

Sample	Raw Reads	Clean Reads	Clean Bases	Q20	Q30	GC Content
AS96hC1	46,080,628	44,799,936	6.72G	97.29%	92.89%	49.98%
AS96hC2	46,016,190	44,600,408	6.69G	97.58%	93.58%	52.14%
AS96hC3	43,874,812	42,545,422	6.38G	97.51%	93.32%	48.84%
AS96hT1	44,745,214	43,474,512	6.52G	97.47%	93.31%	48.98%
AS96hT2	45,812,738	44,663,176	6.70G	97.83%	94.07%	52.66%
AS96hT3	46,227,832	44,959,256	6.74G	97.49%	93.26%	49.18%

## Data Availability

The datasets supporting the results of this article have been submitted to the NCBI Sequence Read Archive (SRA) and accession number shown in the article.

## Supplementary Materials

The following are available online at https://www.mdpi.com/article/10.3390/ani12121517/s1, Table S1. The specific primers used for 10 selected genes in qRT-PCR analysis. Table S2. The information fo the differentially expressed mRNA according to padj < 0.05. Table S3. The information for the differentially enriched GO terms according to *p*-value < 0.05. Table S4. The information for the differentially enriched KEGG terms according to *p*-value < 0.05. Figure S1. KEGG annotation pathway map of the upregulated ECM-receptor interaction signaling pathway. Boxes with red and green borders represent the upregulated differentially expressed mRNAs (DEmRNAs) and downregulated DEmRNAs. Boxes with yellow borders represents containing both upregulated and downregulated DEmRNAs.
