# Supplementary figures and images for: Toxocara canis Infection Alters mRNA Expression Profiles of Peripheral Blood Mononuclear Cells in Beagle Dogs at the Lung Infection Period
Source: Animals (Basel). 2022 Jun 10;12(12):1517. doi: 10.3390/ani12121517 (PMC9219457; doi:10.3390/ani12121517)

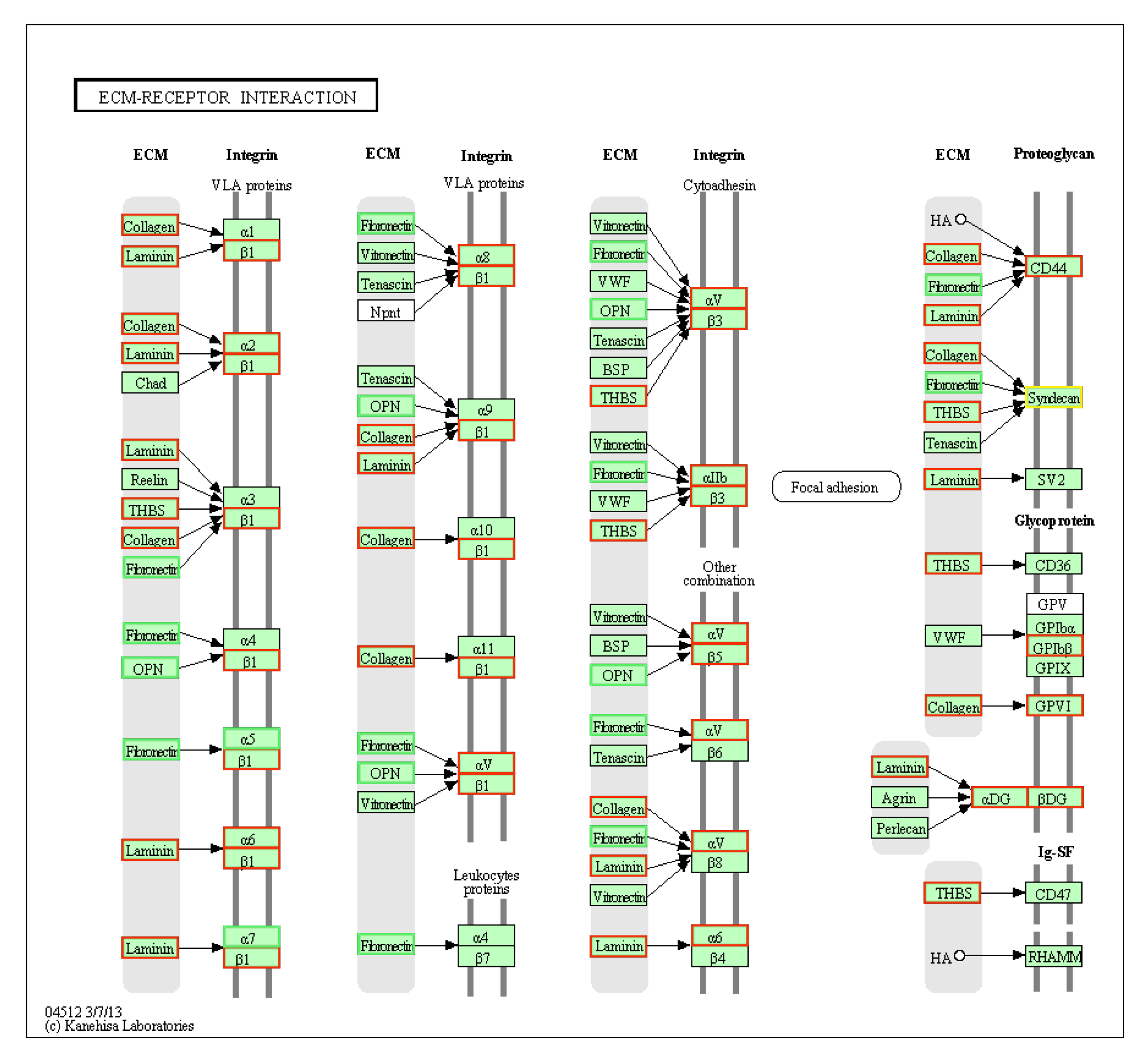

Supplement: Supplementary file 1 [file animals-12-01517-s001.zip › Figure S1.tif]
